# A Complementary Scale of Biased Agonism for Agonists with Differing Maximal Responses

**DOI:** 10.1038/s41598-017-15258-z

**Published:** 2017-11-13

**Authors:** Javier Burgueño, Marta Pujol, Xavier Monroy, David Roche, Maria Jose Varela, Manuel Merlos, Jesús Giraldo

**Affiliations:** 1 Department of Pharmacology, Drug Discovery & Preclinical Development, ESTEVE, Barcelona, Spain; 2 Laboratory of Molecular Neuropharmacology and Bioinformatics, Institut de Neurociències and Unitat de Bioestadística, Universitat Autònoma de Barcelona, 08193 Bellaterra, Spain; 3 Network Biomedical Research Center on Mental Health (CIBERSAM), Madrid, Spain; 4 Universitat Internacional de Catalunya, Faculty of Economics and Social Sciences, 08017 Barcelona, Spain; 5 Centro Singular de Investigación en Medicina Molecular y Enfermedades Crónicas (CIMUS). Universidad de Santiago de Compostela, La Coruña, Spain

## Abstract

Compelling data in the literature from the recent years leave no doubt about the pluridimensional nature of G protein-coupled receptor function and the fact that some ligands can couple with different efficacies to the multiple pathways that a receptor can signal through, a phenomenon most commonly known as functional selectivity or biased agonism. Nowadays, transduction coefficients (log(τ/K_A_)), based on the Black and Leff operational model of agonism, are widely used to calculate bias. Nevertheless, combining both affinity and efficacy in a single parameter can result in compounds showing a defined calculated bias of one pathway over other though displaying varying experimental bias preferences. In this paper, we present a novel scale (log(τ)), that attempts to give extra substance to different compound profiles in order to better classify compounds and quantify their bias. The efficacy-driven log(τ) scale is not proposed as an alternative to the affinity&efficacy-driven log(τ/K_A_) scale but as a complement in those situations where partial agonism is present. Both theoretical and practical approaches using μ-opioid receptor agonists are presented.

## Introduction

G protein-coupled receptors (GPCRs) are membrane receptors responsible for numerous physiological responses in living systems by transducing the signals embodied in the chemical structure of hormones, neurotransmitters and synthetic ligands from outside to inside the cells^1^. Many diseases are associated with an abnormal functioning of these receptors, which makes them one of the most important target families in drug discovery programs^2^.

Plasticity is a property inherent to the flexible nature of proteins which GPCRs make proficient use of for signaling purposes^3^. It is now well established that GPCRs signal not only through G protein-dependent pathways but also through β-arrestin and other accessory proteins^4^. This multiplicity of signaling pathways has led to a collection of pharmacological concepts (pluridimensional efficacy^5,6^, stimulus trafficking^7^, functional selectivity^8,9^, and biased agonism^10^) which all share a common link: the differential modulation that ligands may exert on receptor signaling.

Given that ligands may differentially drive receptor signaling, either potentiating or inhibiting one pathway over others, the issue arises of how to measure functional responses in order to establish reproducible scales for drug comparison^11^. The approach that is currently most commonly used (the log(τ/K_A_) scale) is based on a combination of estimates of efficacy (τ) and affinity (K_A_) to cancel the confounding interacting effects between both parameters^12^. In an attempt to provide new insights on the measurement of receptor signaling bias, this article presents a new scale (the log(τ) scale) that follows from the one described by Kenakin and coworkers to complement it and give extra substance to the classification of compounds’ bias. We use both scales to study a group of μ-opioid receptor ligands as this receptor’s bias behavior has been thoroughly described^13,14^. We show that two ligands’ properties, efficacy and concentration range, play a role in the results yielded by these two scales, allowing for a better classification of compounds and therefore aiding in the initial stages of drug discovery and hopefully in the development of new therapeutic drugs.

The herein described efficacy-driven log(τ) scale is not proposed as an alternative to the widely used affinity&efficacy-driven log(τ/K_A_) scale but as a complement in those situations where partial agonism is present. We argue that using only the log(τ/K_A_) scale is not sufficient for the analysis of biased agonism for compounds with differing maximal responses. In these cases where, according to the operational model^15^, the expression of the maximum response of a given agonist is E_max_ = E_m_ * τ/(1 + τ), which does not include affinity, a note of caution seems opportune. As biased agonism is becoming a reality in routine screening studies, increasingly accurate protocols for biased signaling determination are now necessary.

## Materials and Methods

### Substances

Buprenorphine and fentanyl were supplied by Johnson Matthey (310030 and 310100). Morphine was adquired at Alcaliber. TRV130 was synthesized by Esteve. Finally, endomorphine-2 and Damgo were adquired from Sigma-Aldrich (SCP0133 and E7384).

### G protein-independent pathway: β-arrestin-2 recruitment assay

Chinese hamster ovary (CHO)-K1 cells engineered to co-express the ProLink™ (PK) tagged human μ-opioid receptor and the Enzyme Acceptor (EA) tagged β-Arrestin-2 from DiscoverX were used (93-0213C2). 5000 cells/well were seeded in 20 μL of PathHunter Cell Plating Reagent in 384 well plates. Twenty-four hours later, 5 μl ligands (dissolved in Hanks’ balanced salt solution (HBSS) containing 20 mM Hepes) were added to the plate. Cells were incubated for 90 min at 37 °C. 6 μL of detection reagent (PathHunter Detection Reagent) were then added and the incubation continued at room temperature for 60 min. Luminescence was recorded (integration time of 1 s) in a Tecan Infinite M1000 Pro reader.

### G protein-dependent pathway: Measurement of cAMP responses by Homogeneous Time Resolved Fluorescence

cAMP measurements on CHO-K1 cells that stably express the human μ-opioid receptor (Perkin Elmer ES-542-C) were performed by using a system based on Homogeneous Time Resolved Fluorescence (HTRF). The HTRF cAMP kit from CisBio (62AM4PEJ) was used according to the manufacturer’s recommendation. 2500 cells/well were seeded the day before the experiment in 10 μl of Opti-Mem (Gibco, 11058-021). On the following day, β-funaltrexamine (β-FNX, Sigma Aldrich, O003) was prepared in OptiMem and cells were treated with 5 μl of either concentration of β-FNX (0, 1, 3, 10, 30, 100, 300 nM) for 2 hours. After that time, cells were washed twice with 40 μl of Optimem. 10 μl of Optimem were finally added and cells were left for one hour at 37 °C. Opioid agonists were prepared in Optimem with 3-isobutyl-1-methyl-xanthine (Sigma-Aldrich, I5879-5G) and forskolin (Tocris, 1099) at 0.5 mM and 7.5 μM respectively and 10 μl added to the cells. After 45 min at 37 °C the reaction was stopped by lysing the cells with a mixture of 10 μl of each HTRF detection reagents. Plates were incubated for an additional hour at room temperature and read at 665 nm/620 nm using a RubyStar Plate reader (BMG LabTech). The conditions were followed as described in ref.^16^.

#### Parameter estimation

Curve fitting was performed by using nonlinear least squares regression. The NLIN procedure of SAS statistical package was applied (SAS/STAT 9.2; SAS Institute, Cary, NC, USA). The Gauss iterative method was employed in solving the nonlinear least squares problem. Equation 1 (this article) of the operational model of agonism^15^ was used for affinity and efficacy parameter estimation. It is known that the operational model cannot be applied to fit a single effect/agonist concentration (E/[A]) curve because there is not a single solution for the estimated parameters^12^. In this regard, two different fitting procedures, namely, the receptor inactivation and the comparative methods, were followed depending on the experimental assay performed. For the G protein-dependent cAMP assay, the receptor inactivation method^17^ was used: seven curves for each tested ligand were obtained by varying the concentration (0, 1, 3, 10, 30, 100 and 300 nM, respectively) of the irreversible antagonist β-FNX. Common operational E_m_, n and K_A_ parameters were shared between curves whereas a τ parameter was defined for each β-FNX concentration-dependent curve^15^. The τ parameter corresponding to the curve yielded in the absence of β-FNX was used for the biased agonism analysis. For the G protein-independent β-arrestin-2 recruitment assay the same compounds as in the cAMP assay were used. However, because of the absence of an appropriate irreversible antagonist for the β-arrestin-2 assay, an alternative method was necessary. The comparative method^18^ was considered suitable because the tested compounds behave as partial agonists in the assay. In the comparative method, it is assumed that the maximal response (E_max_) and the slope parameter (m) yielded by a full agonist through the Hill equation (E = E_max_[A]^m^/(A_50_^m^ + [A]^m^)) match, respectively, the operational parameters maximum response of the system (E_m_) and slope parameter (n). Once determined, E_max_ and m can be used as fixed values in Equation 1 (this article) of the operational model for the estimation of K_A_ and τ parameters of partial agonists. Damgo was used as the full agonist in the present study and its curve data were fitted through the Hill equation. The E_max_ and m parameters of Damgo curve were then used as fixed E_m_ and n values in the fitting of the selected compounds under the operational model (Equation 1, this article). In all fitting procedures K_A_ and τ were estimated as logarithms to approximate the assumption of normality distribution^19^.

The two scales compared in the present study are based on log(τ) and log(τ/K_A_) estimates. Parameter estimates for log(τ) and log(K_A_) and their corresponding standard errors were obtained from the nonlinear-regression curve fitting described above. Log(τ/K_A_) was estimated as log(τ) − log(K_A_). Standard errors for log(τ/K_A_) were calculated from the standard errors of log(τ) and log(K_A_) by including the correlation (r) between both parameters because they are not independent properties. Thus, representing log(τ) as x and log(K_A_) as y, the standard error (se) of log(τ/K_A_) was calculated as ${{\rm{se}}}_{{\rm{x}}-{\rm{y}}}=\sqrt{{{\rm{se}}}_{{\rm{x}}}^{2}+{{\rm{se}}}_{{\rm{y}}}^{2}-2{{\rm{rse}}}_{{\rm{x}}}{{\rm{se}}}_{{\rm{y}}}}$. The 95% confidence intervals of log(τ/K_A_) were calculated as ${\rm{I}}{\rm{C}}95{{\rm{ \% }}}_{{\rm{x}}-{\rm{y}}}={\rm{x}}-{\rm{y}}\mp {{\rm{t}}}_{0.025;\nu }{{\rm{s}}{\rm{e}}}_{{\rm{x}}-{\rm{y}}}$, where the value of ν for the degrees of freedom of the Student’s t-function depends on whether the variances of x and y are statistically equal or different (F-Fisher test).

In the calculation of bias through both ΔΔlog(τ)_G-protein, β-arrestin_ and ΔΔlog(τ/K_A_)_G-protein, β-arrestin_ scales we conclude that there is no bias in one or the other when the confidence interval includes zero. However, inasmuch as a collection of compounds is evaluated, the issue of multiple testing appears and a corresponding correction is necessary. The issue of multiple testing was considered by adjusting the significance level through the Holm’s method. To do that we first transformed the IC95% of each of the compounds in a p-value for a t-test with a null hypothesis of μ = 0. Then the p-values for the selected compounds were adjusted according to the Holm’s method. Afterwards, the IC95% were recalculated by adjusting the α value according to the relative position of the previously calculated p-value. This resulted in adjusted IC95% more prone to include the zero value, which parallels the conventional conservative process of multiple testing involving p-values (a lesser propensity to reject the null hypothesis). Due to the statistical consistency of both inference methods, confidence intervals and hypothesis testing produced the same conclusion (biased agonism or not) for each of the compounds.

## Results and Discussion

### The log(τ/K_A_) and log(τ) scales

The two scales for biased agonism we discuss herein are based on the operational model of agonism, presented in a seminal work by Black and Leff^15^. Equation 1 is the general E/[A] equation of the operational model of agonism.

1 $${\rm{E}}=\frac{{{\rm{E}}}_{{\rm{m}}}{{\rm{\tau }}}^{{\rm{n}}}{[{\rm{A}}]}^{{\rm{n}}}}{{({{\rm{K}}}_{{\rm{A}}}+[{\rm{A}}])}^{n}+{{\rm{\tau }}}^{{\rm{n}}}{[{\rm{A}}]}^{{\rm{n}}}}$$

Where E is the pharmacological effect; E_m_, the maximum effect of the system; [A], the concentration of the agonist A; τ, the operational efficacy of A in the receptor system; K_A_, the dissociation constant of A for the receptor; and n, a parameter related with the slope of the E/[A] curves. A value of n equal to 1 yields rectangular hyperbolic E/[A] curves whereas n values greater and lower than 1 allow for steeper and flatter curves than rectangular hyperbola, respectively^15,20^.

Under the operational model of agonism^15^, τ is defined as τ = [R_T_]/K_E_, where [R_T_] represents the total receptor concentration and K_E_ the value of the concentration of agonist-receptor complex, [AR], for half the maximum possible effect, E_m_; in other words, the inverse of K_E_ reflects the intrinsic efficacy of the AR complex. Thus, τ contains both tissue and ligand-receptor efficacy parameters. Moreover, K_A_ is not a thermodynamic equilibrium dissociation constant but a conditional or functional constant. This is because K_A_ does not correspond to an individual equilibrium step, the binding of the agonist to the inactive receptor conformation, but it incorporates, in addition, the receptor conformational change associated with receptor activation. From a molecular perspective, the concept of receptor activation is present in both τ and K_A_ parameters. As it has been shown^15^, τ may reflect the binding of the transducer G protein to the receptor. More precisely, in the presence of GTP and GDP, receptor activation is proportional to the active state of the quaternary complex (AR*G-GDP), with R* indicating the active conformation of the receptor^21^. In addition, K_A_ is a combined expression of the parameter values for the agonist binding to the bare receptor and the receptor conformational change from the inactive (R) to the active (R*) state^22–24^.

The asymptotic maximum (E_max_ in Equation 2) and location (logEC_50_ in Equation 3) parameters allow for the quantification of E/[A] curve shape^20^. LogEC_50_ provides information about the potency of the agonist and E_max_ reflects agonist efficacy. We see that logEC_50_, or its commonly used negative value, pEC_50_, includes operational K_A_ and τ parameters whereas K_A_ is not present in the definition of E_max_.

2 $${{\rm{E}}}_{{\rm{\max }}}=\mathop{{\rm{lim}}}\limits_{{\rm{x}}\to \infty }{\rm{E}}=\frac{{{\rm{E}}}_{{\rm{m}}}}{{\rm{1}}+\frac{{\rm{1}}}{{{\rm{\tau }}}^{{\rm{n}}}}};\,{\rm{with}}\,{\rm{x}}=\,{\rm{log}}[{\rm{A}}]$$

3 $${{\rm{logEC}}}_{{\rm{50}}}=\,{\rm{log}}\,\frac{{\rm{1}}}{{(\frac{{\rm{2}}}{{{{\rm{K}}}_{{\rm{A}}}}^{{\rm{n}}}}+{(\frac{{\rm{\tau }}}{{{\rm{K}}}_{{\rm{A}}}})}^{{\rm{n}}})}^{\frac{{\rm{1}}}{{\rm{n}}}}-\frac{{\rm{1}}}{{{\rm{K}}}_{{\rm{A}}}}};\,{\rm{with}}\,{{\rm{logEC}}}_{50}=\,{\rm{log}}[{\rm{A}}]\,{\rm{for}}\,{\rm{E}}={{\rm{E}}}_{{\rm{\max }}}/2$$

Equation 2 shows that high values of τ are associated with high values of E_max_, which at the limit (full agonists) reach E_m_. On the contrary, low values of τ determine partial agonism: A value of τ as low as one unit makes E_max_ equal to half E_m_. With respect to potency, Equation 3 shows that higher potency values (lower logEC_50_) result from lower K_A_ and higher τ values. Moreover, high values of τ (full agonists) lead to ${{\rm{logEC}}}_{50}=\,{\rm{log}}\,\frac{1}{\frac{{\rm{\tau }}}{{{\rm{K}}}_{{\rm{A}}}}}$.

Kenakin *et al*.^12^, combining the K_A_ and τ parameters of the operational model^15^, defined a parameter designated the transduction coefficient, ${\rm{log}}(\frac{{\rm{\tau }}}{{{\rm{K}}}_{{\rm{A}}}})$, which provides a one-parameter scale able to classify agonists acting through one receptor. They demonstrated that this scale can be transferred between systems with differing receptor densities and, what is more, a ratio of this parameter relative to a reference ligand provides normalization by taking into account the natural bias of the system, and so is useful for comparing experimental and physiological tissues. Precedents of the transduction coefficient can be found in some publications by Ehlert^25–27^, who used either the ε/K_A_ ratio, with ε being intrinsic efficacy, or the τ/K_A_ ratio.

#### Is the Δlog(τ/K_A_) scale sufficient for biased agonism description? Combining the log(τ/KA) and log(τ) scales: A theoretical example

**Figure 1:**
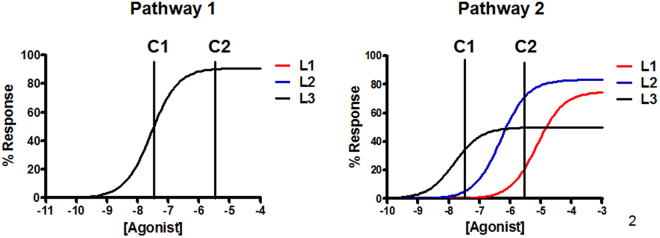
A theoretical example of receptor activation through two different pathways. In the panel on the left it is represented the concentration-response curve of three agonists for a given receptor acting through Pathway 1. In the panel on the right, concentration-response curves are represented for the same agonists acting through Pathway 2. For the sake of clarity, the three agonists show the same effect in Pathway 1 while different behaviors are exerted through Pathway 2. Concentration is the key: Two concentrations are marked (C1 and C2). For both concentrations and in pathway 1, all three ligands show the same response. In pathway 2, differences appear. At concentration C1 the effect observed for agonist 3 is larger than that observed with the two other ligands. However, at concentration C2, the effect of agonist 3 remains greater than that of agonist 1 but smaller than that observed for agonist 2.

As explained in the Appendix (Supplementary Material), both Δlog(τ) and Δlog(τ/K_A_) represent useful scales to classify ligands independently of receptor density. An initial look at both scales reveals the fact that while the former only takes into account ligand operational efficacy, the latter also balances this efficacy in respect to ligand affinity, and so naturally these two scales classify ligands differently and the bias calculated from those scales differs as well. Because the Δlog(τ/K_A_) scale is currently being used in a routine way, the proposal of a complementary scale invites justification. At this point it is worth comparing both scales with a theoretical example:

Let us suppose a drug screening study consisting of two pathways that is aimed at identifying ligands with a positive bias effect of Pathway 2 with respect to Pathway 1. Figure 1 shows the concentration-response curves for three ligands with agonistic properties acting through a given receptor in the two pathways, where the values of τ and K_A_ for each ligand at each pathway are displayed in Table 1. We have assumed that the ligands have the same operational affinities and efficacies in Pathway 1 and different operational affinities and efficacies in Pathway 2. A normalized value of 100 has been assumed for E_m_ in both pathways.

**Table 1:** Operational parameters for the theoretical example.

	Pathway 1					Pathway 2				
	Em	τ	K_A_ (nM)	Δlog(τ/K_A_)	Δlog(τ)	Em	τ	K_A_ (nM)	Δlog(τ/K_A_)	Δlog(τ)
Ligand 1	100	10	300	0	0	100	3	30000	0	0
Ligand 2	100	10	300	0	0	100	5	3000	1.22	0.22
Ligand 3	100	10	300	0	0	100	1	30	2.52	−0.48
				**Bias: Pathway 2 versus Pathway 1**						
					**ΔΔlog(τ/K**_**A**_ **)**	**ΔΔlog(τ)**				
				Ligand1	0	0				
				Ligand2	1.22	0.22				
				Ligand3	2.52	−0.48				

Theoretical values of the operational parameters for three different ligands at a given receptor capable of signaling through two different pathways. Ligand 1 is used as the reference ligand for bias calculation purposes.

For a proper comparison between a collection of ligands within a single pathway and in various pathways, a reference ligand must be defined. This allows for the cancellation of system effects. Assuming Ligand 1 as the reference ligand, calculated parameters for both scales, Δlog(τ) and Δlog(τ/K_A_), at each pathway and the bias of Pathway 2 relative to Pathway 1, ΔΔlog(τ) and ΔΔlog(τ/K_A_), are shown in Table 1. It can be seen that both scales classify ligands in a different order. For the Δlog(τ) scale the order is Ligand 2 > Ligand 1 > Ligand 3 while for the Δlog(τ/K_A_) scale the order is Ligand 3 > Ligand 2 > Ligand 1. Thus, taking Ligand 1 as the reference ligand whose bias is to be optimized, the second scale, Δlog(τ/K_A_), would provide that both Ligand 3 and Ligand 2 are optimized to a greater degree and Ligand 3 to a larger extent than Ligand 2, while with the first scale, Δlog(τ), only Ligand 2 is optimized with respect to Ligand 1.

Another practical output from the combination of the two scales comes from the comparison of the results obtained for Ligand 2 and Ligand 3 in both scales. While for Ligand 2 the two parameters ΔΔlog(τ/K_A_) and ΔΔlog(τ) result in a positive number, suggesting an improvement of the bias with respect to Ligand 1, a different situation is found for Ligand 3. For this last agonist the two parameters show opposing results due to an improvement in affinity but a worsening in efficacy versus Ligand 1.

Although at first sight these results seem contradictory, we understand that the two scales are complementary, each offering information not found in the other. Parameter derivations from the operational model (Equation 1) show that while ΔΔlog(τ/K_A_) is driven by both potency (EC_50_) and affinity (K_A_) (Equation 3) ΔΔlog(τ) is driven by efficacy (E_max_) (Equation 2). A closer look at the concentration-response curves of Pathway 2 in Fig. 1 shows that the concentration at which Ligand 2 and Ligand 3 cross separates the relative activity of both ligands, as explained below.

### Concentration is the key

Following the example above, the agonist concentration at which the E/[A] curves of two agonists at a given pathway cross can be determined.

Let us consider the E/[A] curves of two agonists: A_1_ and A_2_.

4 $${\rm{Agonist}}\,1:\,{{\rm{E}}}_{1}=\frac{{{\rm{E}}}_{{\rm{m}}}{{{\rm{\tau }}}_{1}}^{{\rm{n}}}{[{{\rm{A}}}_{1}]}^{{\rm{n}}}}{{({{\rm{K}}}_{{\rm{A}}1}+[{{\rm{A}}}_{1}])}^{{\rm{n}}}+{{{\rm{\tau }}}_{1}}^{{\rm{n}}}{[{{\rm{A}}}_{1}]}^{{\rm{n}}}}$$

5 $${\rm{Agonist}}\,2:\,{{\rm{E}}}_{2}=\frac{{{\rm{E}}}_{{\rm{m}}}{{{\rm{\tau }}}_{2}}^{{\rm{n}}}{[{{\rm{A}}}_{2}]}^{{\rm{n}}}}{{({{\rm{K}}}_{{\rm{A}}2}+[{{\rm{A}}}_{2}])}^{{\rm{n}}}+{{{\rm{\tau }}}_{2}}^{{\rm{n}}}{[{{\rm{A}}}_{2}]}^{{\rm{n}}}}$$

where it is assumed that E_m_ and n are system-dependent parameters^15^.

The curves for Agonists 1 and 2 cross at the ([A], E) point ([A_1_] = [A_2_] = [A], E_1_ = E_2_ = E). Making Equations 4 and 5 equal and rearranging terms leads to Equation 6.

6 $$[{\rm{A}}]=\frac{{{\rm{\tau }}}_{2}{{\rm{K}}}_{{\rm{A}}1}-{{\rm{\tau }}}_{1}{{\rm{K}}}_{{\rm{A}}2}}{{{\rm{\tau }}}_{1}-{{\rm{\tau }}}_{2}}$$

where [A] is the ligand concentration, τ_1_ and τ_2_ the operational agonist efficacies of Ligand 1 and Ligand 2, respectively, and K_A1_ and K_A2_ the operational agonist equilibrium dissociation constants.

By substituting the value of τ in Equation 6, we obtain Equation 7.

7 $$[{\rm{A}}]=\frac{\frac{[{{\rm{R}}}_{{\rm{T}}}]}{{{\rm{K}}}_{{\rm{E}}2}}{{\rm{K}}}_{{\rm{A}}1}-\frac{[{{\rm{R}}}_{{\rm{T}}}]}{{{\rm{K}}}_{{\rm{E}}1}}{{\rm{K}}}_{{\rm{A}}2}}{\frac{[{{\rm{R}}}_{{\rm{T}}}]}{{{\rm{K}}}_{{\rm{E}}1}}-\frac{[{{\rm{R}}}_{{\rm{T}}}]}{{{\rm{K}}}_{{\rm{E}}2}}}=\frac{{{\rm{K}}}_{{\rm{A}}1}{{\rm{K}}}_{{\rm{E}}1}-{{\rm{K}}}_{{\rm{A}}2}{{\rm{K}}}_{{\rm{E}}2}}{{{\rm{K}}}_{{\rm{E}}2}-{{\rm{K}}}_{{\rm{E}}1}}$$

The ligand concentration ([A]) at which both concentration-response curves cross does not depend upon total receptor concentration ([R_T_]) and it is constant over the whole range of receptor densities.

Looking at the E/[A] curves for Pathway 2 in Fig. 1 we see that Ligand 3 shows greater effect than Ligand 2 at concentrations below that at which both concentration-response curves cross, in agreement with the Δlog(τ/K_A_) scale; while at concentrations above that of curve crossing, Ligand 2 shows the greater effect, in agreement with the Δlog(τ) scale.

Represented again by the same concentration-response curves as in the example above, in Fig. 1 we have also marked two concentrations, C1 and C2, below and above the concentration at which both curves cross in Pathway 2. It can be seen that at C1 the effect of Ligand 3 is greater than that of Ligand 2, and because of this the bias favors Ligand 3 versus Ligand 2. The opposite is true at C2. A similar consideration is exemplified in Kenakin and Christopoulos, 2013 (Fig. 5 in cited article)^22^, where the authors describe how an agonist with a defined calculated bias of one pathway over the other, that is a single value with the Δlog(τ/K_A_) scale, can show variable effective bias *in vivo* in tissues with differing receptor density. In their simulations the authors show how the agonist displays a clear bias throughout the full concentration range in the tissue with a high receptor density. However, in the tissue with low receptor density, the agonist exhibits a change of the preference of one pathway over the other at the ligand concentration at which the E/[A] curves for the two pathways cross. Therefore, for this particular ligand in these particular *in vivo* conditions, the Δlog(τ/K_A_) scale does not reflect correctly the experimental results. The approximation presented here, a joint consideration of the Δlog(τ) and Δlog(τ/K_A_) scales aids in identifying experimentally-found differences, provides better fine tuning of the classification of compounds and allows for the calculation of a concentration value that determines the relationship between the scales.

**Figure 2:**
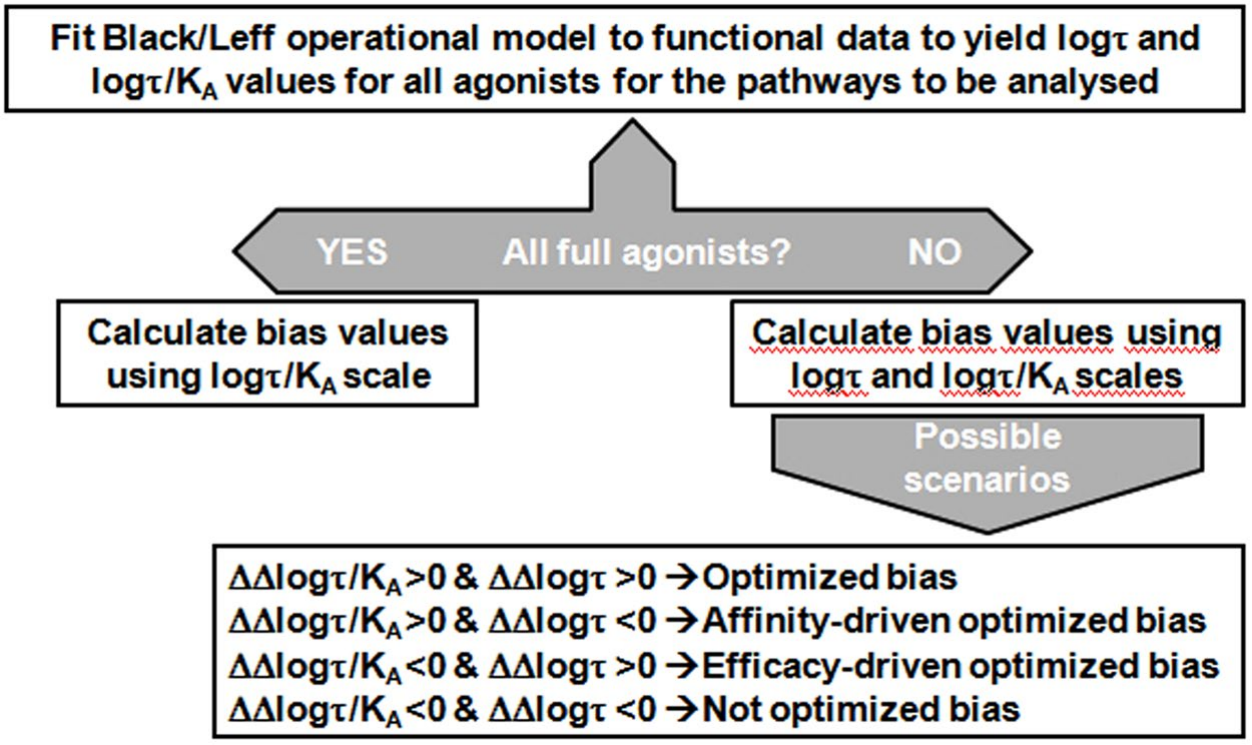
Schematic diagram for analysis of agonist bias using log(τ) and log(τ/K_A_) scales. Properly designed functional data for the pathways to be studied are fitted to Black & Leff operational model to yield log(τ) and log(τ/K_A_) values. If all agonists behave as full agonists in all pathways then log(τ/K_A_) scale alone can be used to classify compounds. If not, then both scales should be used. As a result, four different scenarios may result.

Figure 2 shows a diagram for the analysis of agonist bias using the Δlog(τ) and Δlog(τ/K_A_) scales calculated by fitting functional data to the Black and Leff operational model^15^. If all agonists studied behave as full agonists in all the pathways analyzed, then Δlog(τ/K_A_) scale alone can be used. But, if those agonists are not all full agonists, then both scales should be used, ending up with four different situations. The first situation, where both scales show an improvement in the bias they calculate (ΔΔlog(τ) and ΔΔlog(τ/K_A_) > 0) meaning that bias has been optimized. A second one where ΔΔlog(τ/K_A_) > 0 but Δlog(τ) < 0 would represent a situation where only the first scale would point to an improvement in the bias pursued. As the difference between both scales resides in the K_A_ value of the first one, we identify this bias improvement as affinity-driven, due to an increase in affinity (K_A_) of the ligand studied compared to the reference ligand. A third scenario, where ΔΔlog(τ/K_A_) < 0 but Δlog(τ) > 0 would represent a situation where only the second scale points to an improvement in bias. In this case we identify this bias improvement as efficacy-driven due to an increase in efficacy (τ) of the ligand studied versus the reference. Finally, in the last situation both scales point out that bias improvement has not been achieved (ΔΔlog(τ) and ΔΔlog(τ/K_A_) < 0).

### A practical example

Biased signaling has already been analyzed for the μ-opioid receptor^13,28^ (see also^29^ as a review). What is more, TRV130, a ligand described as a biased μ-opioid agonist favoring the G protein signaling pathway over that of β-arrestin, is already in Phase II clinical trials^30^. Bearing this in mind, we used the μ-opioid receptor to apply our proposal for bias calculation.

When µ-opioid receptors couple to Gi/o subtypes they inhibit the production of cAMP and they can also recruit β-arrestins. An HTRF (Cisbio) cAMP determination assay was used to determine the activity of this receptor on the G protein signaling pathway, while an enzyme complementation assay (DiscoverX) was used to determine its ability to recruit and signal through the β-arrestin pathway.

The ligands used in this study were: morphine and fentanyl, two opioids commonly used for pain-relief; buprenorphine, a classically classified partial agonist; and finally endomorphine-2 and TRV130, which are biased agonists for the μ-opioid receptor^13,31^.

**Figure 3:**
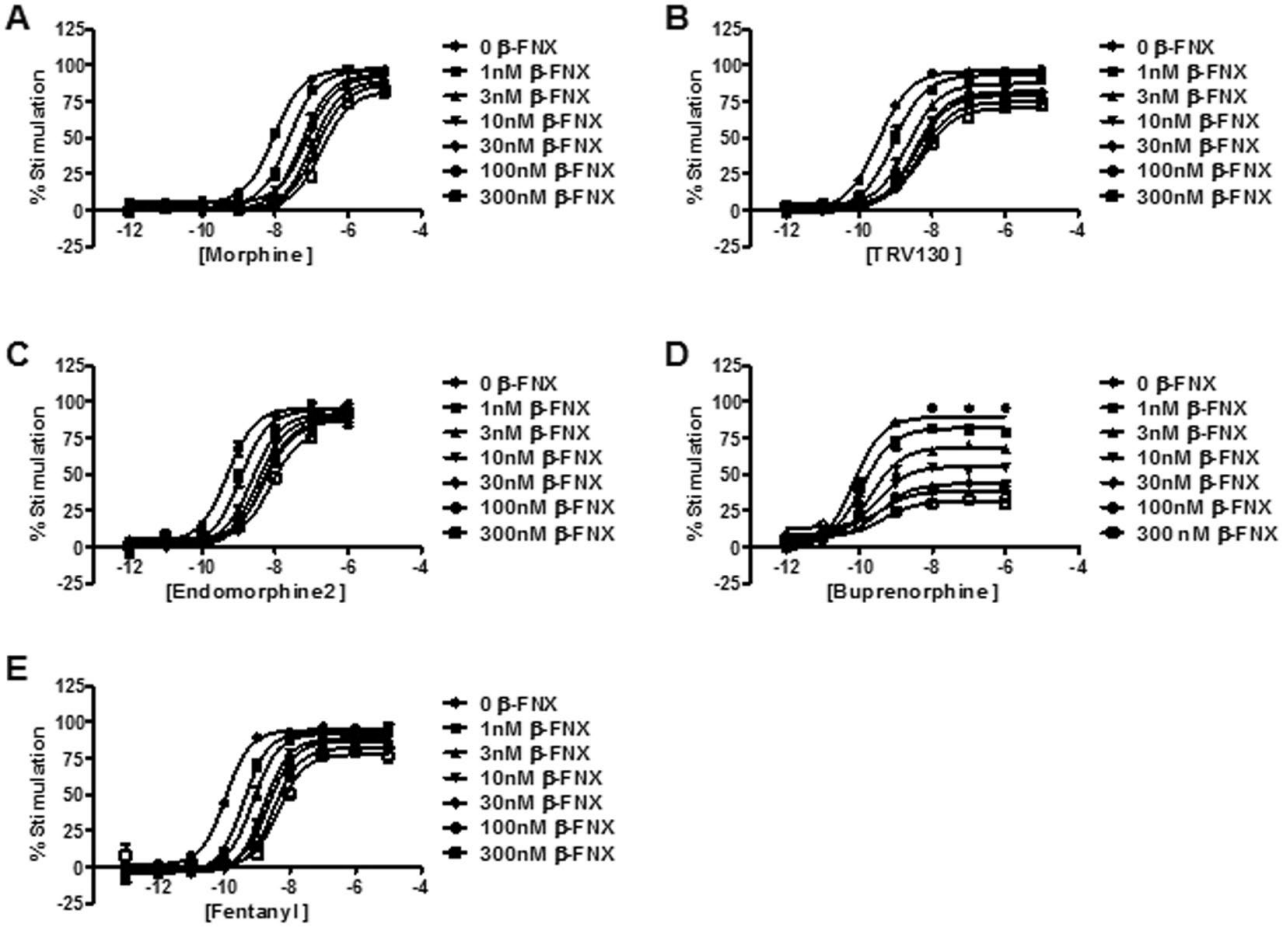
μ-opioid agonist inhibition of forskolin-stimulated cAMP production assay (G protein-dependent pathway). The concentration-response curves for five different opioid agonists were determined in both the absence and presence (1, 3, 10, 30, 100, 300 nM) of the irreversible antagonist β-funaltrexamine. (**A**) Morphine; (**B**) TRV130; (**C**) Endomorphine2; (**D**) Buprenorphine; (**E**) Fentanyl. Results were obtained in at least three independent experiments. In each experiment, data points were obtained in duplicates.

Figure 3 shows the results of the five μ-opioid receptor agonists in the cAMP determination assay. It is known^32^ that the operational model cannot satisfactorily fit a single experimental E/[A] curve. A solution to the problem can be reached by using the irreversible inactivation method^17^ (see Parameter estimation in Methods), which produces a collection of experimental curves with lower maximal effect by decreasing receptor density. With this procedure, a single solution for each of the curves, with particular τ and common E_m_, n and K_A_ parameters, is obtained. We followed this approach for parameter determination in the G protein pathway. Results are shown in Table 2.

**Table 2:** Operational parameters (estimates ± standard errors) for the G protein-dependent pathway.

	E_m_	n	Log (K_A_)	Log (τ)	Log(τ/K_A_)
Morphine	98.33 ± 1.41	1.04 ± 0.05	−5.96 ± 0.11	2.12 ± 0.12	8.08 ± 0.04
Fentanyl	93.99 ± 1.01	1.22 ± 0.08	−7.74 ± 0.10	2.23 ± 0.11	9.97 ± 0.05
TRV130	98.15 ± 1.44	0.90 ± 0.04	−7.63 ± 0.07	1.82 ± 0.09	9.45 ± 0.05
Endomorphine-2	96.64 ± 2.12	1.02 ± 0.07	−7.38 ± 0.17	1.97 ± 0.20	9.35 ± 0.07
Buprenorphine	96.75	0.98 ± 0.21	−9.35 ± 0.23	0.85 ± 0.20	10.20 ± 0.10

Data obtained from concentration-response curves of µ-opioid agonists in presence of various concentrations of the irreversible antagonist β-FNX analyzed with the operational model of agonism (Fig. 3). Parameter estimates and standard errors of operational parameters E_m_, n, log(K_A_) and log(τ) were produced by global fitting. Common E_m_, n and K_A_ parameters were shared between curves whereas a τ parameter was defined for each β-FNX concentration-dependent curve. In the Table, log(τ) for β-FNX concentration equal to 0 is shown. For buprenorphine, the fitting did not converge when E_m_ was included as a free parameter; thus, we set E_m_ equal to the mean of the values obtained for the other ligands (96.75) and kept it fixed as such in the fitting process. Log (τ/K_A_) values and their standard errors were calculated from estimated τ and K_A_ parameters (see *Parameter estimation* in Methods).

**Figure 4:**
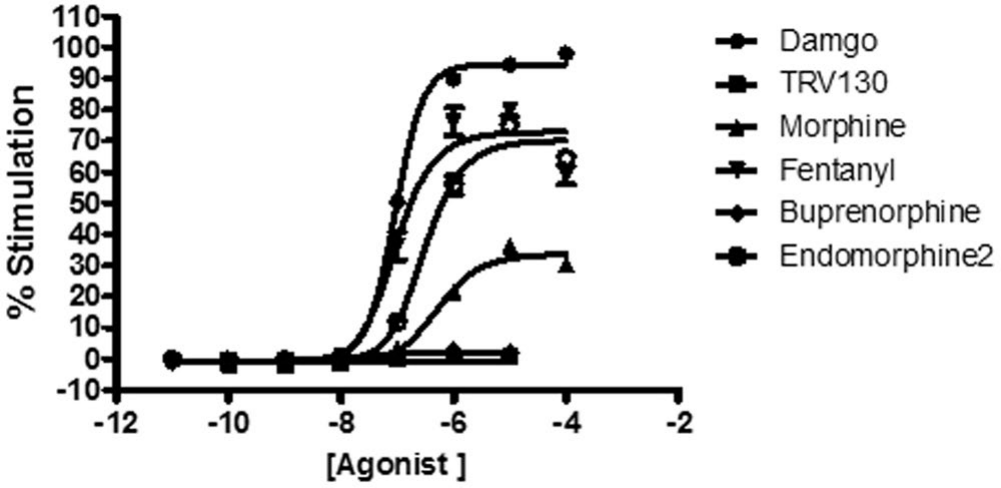
μ-opioid agonist β-arrestin recruitment assay. The concentration-response curves for five different opioid agonists were determined and compared to the concentration-response curve of damgo as the standard full agonist for this assay. Results were obtained in at least three independent experiments. In each experiment, data points were obtained in quadruplicates.

For the β-arrestin pathway (Fig. 4) we used a different experimental approach: the comparative method^18^ (see Parameter estimation in Methods). In this method, it is assumed that the maximal response (E_max_) and the slope parameter (m) yielded from a full agonist by fitting curve data with the Hill equation match, respectively, the operational E_m_ and n parameters, and, once determined, can be used as fixed values for the estimation of the efficacy and affinity of partial agonists within the operational model. In this assay, we used Damgo as the full agonist. As all the other opioids in the assay behaved as partial agonists, their K_A_ and τ values could be directly calculated using the operational model by substituting E_m_ and n parameters with Damgo E_max_ and m and keeping them fixed as such in the fitting process. Results are shown in Table 3. It is worth noting the discrepancies in K_A_ between the two pathways for each of the agonists (Tables 2 and 3). This is an acceptable result under the operational model of agonism because K_A_ is a functional affinity of the agonist which includes the interaction of the activated receptor with the signaling protein either the G protein or β-arrestin^22^.

**Table 3:** Operational parameters (estimates ± standard errors) for β-arrestin pathway.

	Log (K_A_)	Log (τ)	Log (τ/K_A_)
Morphine	−6.31 ± 0.10	−0.19 ± 0.02	6.12 ± 0.09
Fentanyl	−6.70 ± 0.19	0.35 ± 0.08	7.05 ± 0.15
TRV130	−6.96 ± 0.99	−1.11 ± 0.15	5.85 ± 0.92
Endomorphine-2	−6.90 ± 0.08	0.29 ± 0.03	7.19 ± 0.06
Buprenorphine	−7.49 ± 0.30	−1.09 ± 0.04	6.40 ± 0.28

Data obtained from concentration-response curves of µ-opioid agonists using the comparative method^18^ with Damgo as full agonist (Fig. 4). The Hill equation was used for fitting to Damgo data. The values obtained for Damgo for maximal response (95.14) and slope parameter (1.48) were used for all ligands in the Table as E_m_ and n parameters in the operational model and kept fixed as such in the fitting process. Parameter estimates and standard errors of operational parameters log(K_A_) and log(τ) were produced by global fitting. Log (τ/K_A_) values and their standard errors were calculated from estimated τ and K_A_ parameters (see *Parameter estimation* in Methods).

**Figure 5:**
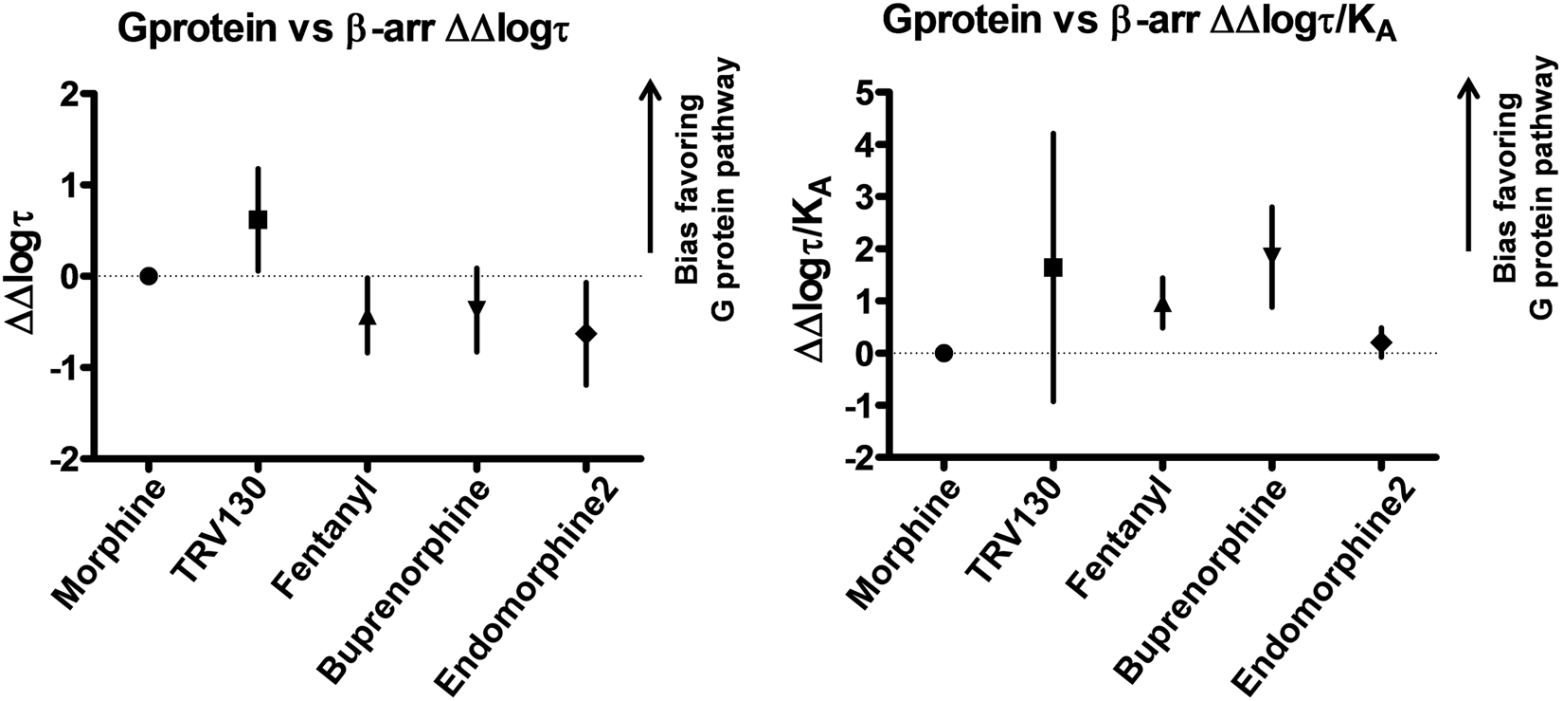
Graphical representation of ΔΔlog(τ) and ΔΔlog(τ/K_A_) scales. The left panel represents ligand bias using ΔΔlog(τ) values of all ligands referred to morphine as the common reference ligand for cAMP inhibition versus β-arrestin interaction. Similar results are shown in the right panel using the ΔΔlog(τ/K_A_) scale.

At this point it is worth mentioning, for the sake of correct data interpretation, that it is convenient to analyze all the studied pathways in the same cell line to minimize any functional influence of receptor tagging or modification that needs to be performed. Unfortunately, this is not always possible because, depending on the signaling pathway, different receptor or signaling protein constructs must be used^33^. This is particularly evident when more than two pathways are analyzed, as in the study by Thompson and colleagues^28^, where bias was calculated for cAMP, GTPγS and pERK1/2 determinations using the wild-type μ-opioid receptor, but for other signaling pathways such as β-arrestin-1, β-arrestin-2 or receptor internalization an Rluc-tagged receptor was used^28^. It is worth noting that concerns about receptor tagging have been extensively addressed in the literature. Barak and colleagues, back in 1997^34^, described a β_2_-adrenoceptor variant tagged with eGFP at its C-terminal part which showed ligand binding, second messenger stimulation, receptor phosphorylation and internalization properties closely resembling those of the wild-type receptor. In another example, Scherrer and colleagues compared an eGFP tagged δ-opioid receptor with its wild-type counterpart with both transfected in HEK293 cells^35^. They showed that the binding of different opioid ligands remained the same between the two receptors and more importantly there was no difference in the capacity of the deltorphin II agonist to stimulate the receptor as measured by using a [^35^S]-GTPγS binding assay. A final example can be found in the orphan GPR17 receptor^36^. In this case, a label-free dynamic mass redistribution assay was used to compare the functional response of the wild-type receptor with that of an N-terminal hemagglutinin-tagged receptor, as well as with either a C-terminal Rluc-tagged or a C-terminal GFP^2^-tagged receptor. Also, the ability of the wild-type receptor to stimulate cAMP response was compared with that of the C-terminal GFP^2^-tagged receptor. In all cases, the introduction of the corresponding tag had no effect on receptor functionality. Finally, the main concern when using different cells or differently tagged receptors is that system bias and observational bias may differ between the different cell systems used. To address these issues and cancel out both system and observational bias, which can be expected to affect all agonists to the same extent, all bias factors are related to a common reference agonist^12,22^. In the present work, to calculate bias factors between the two studied pathways, morphine was selected as the reference agonist. The calculation of bias involves parameters (τ and K_A_) which were estimated as logarithms for reasons of normality. Thus, bias evaluation implies the calculation of differences in logarithmic values and the estimation of their corresponding confidence intervals (see Parameter estimation in Methods). Bias estimates of the (G protein-β-arrestin) − ΔΔlog(τ) and −ΔΔlog(τ/K_A_) scales for the five opioids used in this study are shown in Table 4 and represented in Fig. 5. As can be seen in the right panel of this figure, all the ligands show a positive bias compared to morphine in the ΔΔlog(τ/K_A_) scale, though not statistically significant (zero is included in the confidence interval) in the case of TRV130 and endomorphine-2. On the contrary, when analyzing the data in the ΔΔlog(τ) scale (Fig. 5 left panel), a different picture is obtained. In this case, only TRV130 shows a clear positive bias favoring the G protein effect versus that of β-arrestin. Fentanyl, endomorphine-2 and buprenorphine show bias favoring the β-arrestin pathway, though not statistically significant in the last case.

**Table 4:** Calculation of (G protein - β-arrestin) ΔΔlog(τ) and ΔΔlog(τ /K_A_) bias.

	ΔLog (τ) G protein pathway	ΔLog (τ) β-arrestin pathway	ΔΔLog (τ) G protein – β arrestin	ΔLog(τ/K_A_) G protein pathway	ΔLog(τ/K_A_) β-arrestin pathway	ΔΔLog(τ/K_A_) G protein – β arrestin
Morphine	0	0	0	0	0	0
Fentanyl	0.11 ± 0.16	0.54 ± 0.08	−0.43 ± 0.18 (−0.84, −0.02) *	1.89 ± 0.06	0.93 ± 0.19	0.96 ± 0.20 (0.48, 1.44) *
TRV130	−0.3 ± 0.15	−0.92 ± 0.15	0.62 ± 0.21 (0.06, 1.18) *	1.37 ± 0.06	−0.27 ± 0.92	1.64 ± 0.92 (−0.93, 4.21)
Endomorphine-2	−0.15 ± 0.23	0.48 ± 0.04	−0.63 ± 0.23 (−1.19, −0.07) *	1.27 ± 0.08	1.07 ± 0.11	0.20 ± 0.14 (−0.08, 0.48)
Buprenorphine	−1.27 ± 0.23	−0.9 ± 0.04	−0.37 ± 0.23 (−0.83, 0.09)	2.12 ± 0.11	0.28 ± 0.29	1.84 ± 0.38 (0.88, 2.80) *

Raw data for Log (τ) and log(τ /K_A_) were taken from Tables 2 and 3. Morphine was taken as the reference compound. Parameter estimates ± standard errors are shown. The confidence intervals of 95% (CI95%) for ΔΔ estimates are shown in parentheses. Multiple testing was considered in the calculation of confidence intervals through Holm’s method (see *Parameter estimation* in Methods). A * has been added to those CI95% for ΔΔ estimates which do not include zero and show thus statistical significance for bias signaling.

DeWire and colleagues (2013)^14^ reported a bias favoring the G protein pathway for TRV130 using relative intrinsic activities (RA_i_) and because RA_i_ values can reduce to τ/K_A_ (when n = 1)^12^, their data resembles the transduction coefficient scale (ΔΔlog(τ/K_A_)) that we present here. Regarding endomorphine-2, some studies^13,37^ have reported a bias favoring the β-arrestin pathway for this ligand. In these studies^13,37^, τ values were estimated from the operational model using K_A_ values obtained from independent binding experiments. These results for endomorphine-2 favoring the β-arrestin pathway are in agreement with our results in the ΔΔlog(τ) scale, though in our fitting procedure τ and K_A_ are both estimated from the operational model.

Quantitative pharmacology of signaling bias may offer a structure-function framework which can be useful for drug discovery purposes. An elegant study on the M_2_ muscarinic acetylcholine receptor combining various approaches including mutation and molecular modeling identified orthosteric and allosteric site mutations that contribute to ligand-selective signaling bias^38^. The authors suggested that the functional selectivity of some of the compound might arise from a bitopic mechanism^38^. Other examples with similar methodological approaches can be cited as, for example, studies focusing on the glucagon-like peptide-1 receptor^39^ or the M_1_ muscarinic acetylcholine receptor^40^. Moreover, a detailed review on the functional analysis of receptor states can be found in ref.^21^.

**Figure 6:**
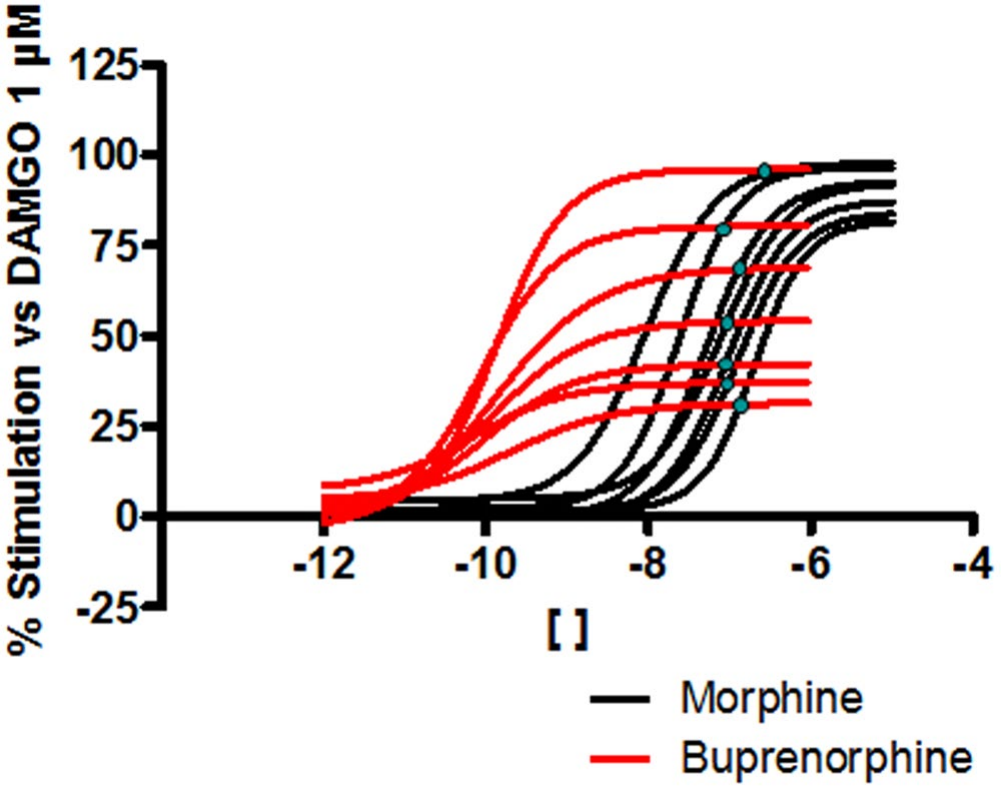
μ-opioid agonist inhibition of forskolin-stimulated cAMP production assay. The concentration-response curves for morphine and buprenorphine determined in both the absence and presence (1, 3, 10, 30, 100, 300 nM) of the irreversible antagonist β-funaltrexamine are represented jointly in the same graph. The concentrations at which the curves for each agonist cross with each other are marked. Results were obtained in at least three independent experiments. In each experiment, data points were obtained in quadruplicates.

In the theoretical example used, we determined the concentration at which the concentration-response curves of two agonists for a given receptor cross each other at a given signaling pathway. We showed that this concentration does not depend on the total amount of receptor present. In Fig. 6 we have represented the concentration-response curves for morphine and buprenorphine in the cAMP inhibition assay in the presence or absence of β-FNX to illustrate this point. Visual inspection of Fig. 6 shows that the concentration-response curves of morphine and buprenorphine at various β-FNX concentrations cross at similar concentration values (shown by blue dots), in agreement with theoretical predictions (Equation 7). Applying Equation 6 to the data generated from these two agonists gives the following concentration values (critical concentrations) at which the curves cross: 80 nM, 102 nM, 124 nM, 90 nM, 82 nM, 71 nM and 88 nM, respectively, for each of the β-FNX pretreatment conditions. We see that the effect elicited by buprenorphine is always greater than that produced by morphine at concentrations lower than ~100 nM whereas the opposite is true at concentrations above ~100 nM.

## Conclusions

Biased agonism is a hot topic in current pharmacologic research with known therapeutic implications. Accurate and standardized measurement of this property is fundamental to drug discovery and development. Currently, the most widely used scale is one based on log(τ/K_A_). It has the advantage of combining efficacy and affinity properties in a single parameter thus providing simplicity. However, in those situations in which different maximal responses are found, the log(τ/K_A_) scale appears to be insufficient. In this regard, because the efficacy parameter τ is directly related with the maximal response achieved by an agonist, the log(τ) scale can complement the log(τ/K_A_) scale in those cases which include ligands with different maximal responses. Of note, we have shown that the log(τ) scale accomplishes the same requirement as that of the log(τ/K_A_) scale, namely the ratio of τ values for two ligands across receptor systems with varying receptor density remains constant. We have also shown that concentration plays a role in these cases and how the decision of whether to use a biased agonism approach based on either pure efficacy (the ΔΔlog(τ) scale) or a combination of efficacy and affinity parameters (the ΔΔlog(τ/K_A_) scale) depends on the experimental concentration window used. In this regard, the signs of the (ΔΔlog(τ/K_A_), ΔΔlog(τ)) pairs provide an indication on whether there is (+, +) or not (−, −) an optimization of the bias in one pathway relative to the other also whether it is mainly affinity- or efficacy-driven, (+, −) and (−, +), respectively. Finally, we have illustrated the application of the proposed methodology to the μ-opioid receptor scenario by considering the G protein and the β-arrestin pathways and selected full and partial agonists.

## Electronic supplementary material

Supplementary Information
