# Supplementary material for: A Complementary Scale of Biased Agonism for Agonists with Differing Maximal Responses
Source: Sci Rep. 2017 Nov 13;7:15389. doi: 10.1038/s41598-017-15258-z (PMC5684405; doi:10.1038/s41598-017-15258-z)
Supplement: Supplementary file 1 — Supplementary Information [file 41598_2017_15258_MOESM1_ESM.pdf]

A Complementary Scale of Biased Agonism for Agonists with Differing Maximal Responses

Javier Burgueño[1], Marta Pujol[1], Xavier Monroy[1], David Roche[2,3], Maria Jose Varela[4], Manuel Merlos[1] and Jesús Giraldo[2*]

[1]Department of Pharmacology,
Drug Discovery & Preclinical Development,
ESTEVE, Barcelona, Spain.
[2]Laboratory of Molecular Neuropharmacology and Bioinformatics,
Institut de Neurociències and Unitat de Bioestadística,
Universitat Autònoma de Barcelona, 08193 Bellaterra, Spain; Network Biomedical Research Center on Mental Health (CIBERSAM)
[3]Universitat Internacional de Catalunya, Faculty of Economics and Social Sciences, 08017 Barcelona, Spain
[4]Centro Singular de Investigación en Medicina Molecular y Enfermedades Crónicas (CIMUS). Universidad de Santiago de Compostela. Spain

Appendix

$\Delta\log(\tau)$ does not depend on receptor density

Following the rationale of Kenakin *et al.*[1] of taking two ligands in two different conditions, where the total receptor densities ($[R_T]$) have changed between each condition, for instance by receptor alkylation (Appendix Figure A1), two conditions are established:

Condition 1: $$\Delta\log\left(\frac{\tau}{K_A}\right)_1 = \log\left(\frac{\tau_{11}}{K_{A1}}\right) - \log\left(\frac{\tau_{21}}{K_{A2}}\right) \qquad \text{(A1)}$$

Where $\Delta\log\left(\frac{\tau}{K_A}\right)_1$ stands for the difference between two ligands in condition 1, with τ_{i1} defined as the operational efficacy of either ligand 1 or 2 in condition 1 and K_{Ai}, the dissociation constant of either ligand 1 or 2. No sub-index for condition is included in K_A because this parameter does not depend on the total receptor concentration. Yet, it is a ligand-receptor parameter.

Condition 2: $$\Delta\log\left(\frac{\tau}{K_A}\right)_2 = \log\left(\frac{\tau_{12}}{K_{A1}}\right) - \log\left(\frac{\tau_{22}}{K_{A2}}\right) \qquad \text{(A2)}$$

Where, analogously to condition 1, $\Delta\log\left(\frac{\tau}{K_A}\right)_2$ stands for the difference between two ligands in condition 2, with τ_{i2} defined as the operational efficacy of either ligand 1 or 2 in condition 2 and K_{Ai}, the dissociation constant of either ligand 1 or 2. It is worth noting that this $\Delta\log\left(\frac{\tau}{K_A}\right)$ scale can also be found in Ref. 2, where it was used for estimating the selectivity of agonists for M1 to M4 muscarinic receptor subtypes.

Kenakin et al.[1] compared three scales for agonist activity, which were based on (i) pEC_{50}, (ii) $\log(RA) = \log\left(\frac{E_{max}}{EC_{50}}\right)$, and (iii) $\log\left(\frac{\tau}{K_A}\right)$ parameters. The authors found, by performing simulations with the operational model, that $\Delta\log(\tau/K_A)$ between two

agonists, but not the other two scales, is stable to changes in receptor concentration (Eq. A3) for any value of the slope parameter n (Eq. 1 main text).

$$\Delta \log\left(\frac{\tau}{K_A}\right)_1 = \Delta \log\left(\frac{\tau}{K_A}\right)_2 \qquad \text{(A3)}$$

Thus, it follows that:

$$\log\left(\frac{\tau_{11}}{K_{A1}}\right) - \log\left(\frac{\tau_{21}}{K_{A2}}\right) = \log\left(\frac{\tau_{12}}{K_{A1}}\right) - \log\left(\frac{\tau_{22}}{K_{A2}}\right) \qquad \text{(A4)}$$

$$\log \tau_{11} - \log K_{A1} - \log \tau_{21} + \log K_{A2} = \log \tau_{12} - \log K_{A1} - \log \tau_{22} + \log K_{A2} \qquad \text{(A5)}$$

$$\log \tau_{11} - \log \tau_{21} = \log \tau_{12} - \log \tau_{22} \qquad \text{(A6)}$$

$$\Delta \log(\tau)_1 = \Delta \log(\tau)_2,\ \log\left(\frac{\tau_{11}}{\tau_{21}}\right) = \log\left(\frac{\tau_{12}}{\tau_{22}}\right),\ \frac{\tau_{11}}{\tau_{21}} = \frac{\tau_{12}}{\tau_{22}} \qquad \text{(A7)}$$

Equation (A7) shows that the ratios of τ values for two given ligands also remain constant and are not dependent upon either receptor density or the ratios of τ/K_A values. Of note, the constant value of the ratio of τ values for two ligands across receptor systems with varying receptor density is a theoretical consequence of τ definition. So, this $\Delta\log(\tau)$ represents an alternative scale to classify ligands with a different relevancy than that of the $\Delta\log(\tau/K_A)$ scale as seen below. It is worth mentioning that although $\log(\tau)$ scale resembles one proposed by Rajagopal and coworkers[3] (effective signaling factor (σ_{lig})) the main difference between them is that we do not assume a singular K_A for all functional pathways analyzed, with this affinity value obtained by an independent binding assay. Rather, τ and K_A are estimated by the operational model allowing both parameters to vary between pathways. Thus, our proposal does not imply using different methodologies for obtaining either τ/K_A or τ values but defining two scales whose parameters are obtained with the same experimental and computational procedures.

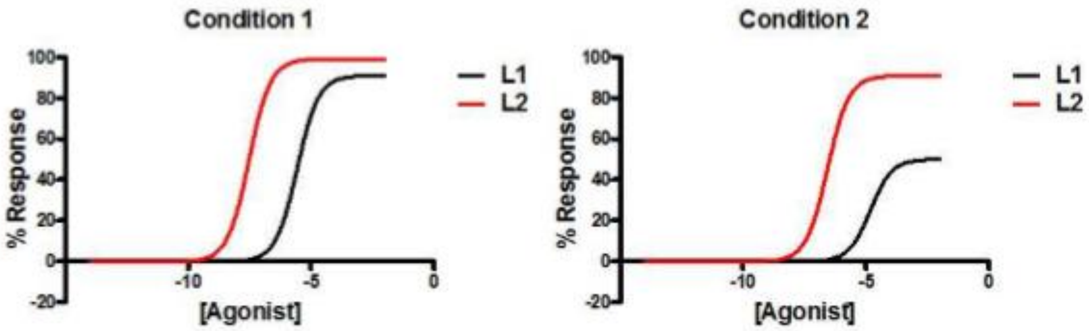

Figure A1. A theoretical example of receptor alkylation. In the panel on the left it is represented the concentration-response curves of two agonists (L1 and L2) for a given receptor in a condition where $[R_T] = [R_T]_1$. In the panel on the right the concentration response curves of the same ligands are represented in a condition where $[R_T] = [R_T]_2$. Condition 2 represents a situation where total receptor concentration has been reduced by alkylation, $[R_T]_1 > [R_T]_2$.

References

1. Kenakin, T., Watson, C., Muniz-Medina, V., Christopoulos, A. & Novick, S. A simple method for quantifying functional selectivity and agonist bias. ACS Chem. Neurosci. 3, 193-203 (2012).
2. Figueroa, K. W., Griffin, M. T. & Ehlert, F. J. Selectivity of agonists for the active state of M1 to M4 muscarinic receptor subtypes. J. Pharmacol. Exp. Ther. 328, 331-342 (2009).
3. Rajagopal, S., Ahn, S., Rominger, D. H., Gowen-MacDonald, W., Lam, C. M., DeWire, S. M. et al. Quantifying ligand bias at seven-transmembrane receptors. Mol. Pharmacol. 80, 367-377 (2011).
